# Supplementary material for: Toll-like receptors 1–9 in small bowel neuroendocrine tumors–Clinical significance and prognosis
Source: PLoS One. 2024 May 6;19(5):e0302813. doi: 10.1371/journal.pone.0302813 (PMC11073674; doi:10.1371/journal.pone.0302813)
Supplement: Attachment — Submitted filename: (PONE-D-23-35599) Review comments.docx [file pone.0302813.s002.docx]

1. The manuscript (PONE-D-23-35599) entitled Toll-like receptors 1–9 in small bowel neuroendocrine tumors – Clinical significance and prognosis' has been carefully reviewed as per the guidelines of the journal. In my point of view, the manuscript is judged to be of potential interest to the readers and the scientific community as well. While the overall concept of the paper is reasonably satisfactory, it falls within the scope of the journal. However, it has some corrections. The manuscript still has a number of flaws, which need to be addressed before it is formally accepted for publication. For instance, there are some errors in the manuscript's scientific, typographical, and grammatical content. Overall, this manuscript may offer important contributions to the literature, but needs some minor corrections for possible publication in this journal
2. Title is Ok
3. Abstract: the abstract is factual concise and explain briefly the purpose of the research. However it would be better to add some key results related insights in the section as well. Also I would recommend suggesting some implications from the finding to represent future perspectives of the study.
4. Introduction: the introduction part is written beautifully, precise and meet the theme of the work.
5. Methods: This section is also written very clear.
6. Results: All results are ok
7. Discussion: this part is not very clear, the author should touch every part of the results and compare results with already published work with solid reasons.
8. Conclusion: In conclusion please summarize the study’s main findings or insights you want to convey as a result of this MS. The limitations and strengths should primarily be highlighted, which could be very helpful in determining the future direction in this field of research direction.
9. Tables: In the tables what does mean by class M, N and class T? You would better explain it the methods and results.
10. Figures: Figures 1 part B, C, E and F are not clear, please provide high resolution clear images.
11. References: Crosscheck all references and arrange them in accordance with the guidelines of the journal.
