# Supplementary material for: Toll-like receptors 1–9 in small bowel neuroendocrine tumors–Clinical significance and prognosis
Source: PLoS One. 2024 May 6;19(5):e0302813. doi: 10.1371/journal.pone.0302813 (PMC11073674; doi:10.1371/journal.pone.0302813)
Supplement: Attachment — Submitted filename: Response to Reviewers.docx [file pone.0302813.s003.docx]

Dr. Mohsan Ullah
Academic Editor
PLOS ONE
PONE-D-23-35599

Dear Dr. Ullah,

We are pleased to submit our revision of the manuscript PONE-D-23-35599 titled “Toll-like receptors 1–9 in small bowel neuroendocrine tumors – Clinical significance and prognosis”. We thank for the review and insightful comments. Point-to-point description of the changes made to the manuscript are provided below.

The updated Financial Disclosure Statement was also asked to be included in this cover letter. There was also a notion in the comments, about the grant information not matching under ‘Funding Information’ and ‘Financial Disclosure’ sections. The updated Financial Disclosure Statement can be found below. The Funding Information section of the submission form was checked to match with the Financial Disclosure Statement. Funder URLs are now included. Only The Finnish Medical Foundation used a specific grant number, which has now been added in the Financial Disclosure Statement and Funding information sections.

Updated Financial Disclosure Statement: This study was funded by The Finnish Medical Foundation grant no. 5954 (NH), Mary and Georg C. Ehrnrooth Foundation (NH, OH), Instrumentarium Science Foundation (OH) and Finnish State Research Funding (OH). The funders had no role in study design, data collection and analysis, decision to publish, or preparation of the manuscript.

<https://laaketieteensaatio.fi/en/home/>

<https://www.instrufoundation.fi/en.php>

<https://marygeorg.fi/en/home/>

<https://valtioneuvosto.fi/en/frontpage>

We hope that our revised manuscript will be accepted for publication in PLOS ONE.

Yours sincerely,

Niko Hiltunen, MD
Translational Medicine Research Unit, Medical Research Center, University of Oulu and Oulu University Hospital, Oulu, Finland
Aapistie 5, 90220 Oulu
FINLAND
E-mail: [niko.hiltunen@oulu.fi](mailto:niko.hiltunen@oulu.fi)

January 22nd, 2024
Manuscript: PONE-D-23-35599
Title: Toll-like receptors 1–9 in small bowel neuroendocrine tumors – Clinical significance and prognosis

**Authors’ responses to the Academic Editor’s comments** (Academic Editor’s comments are reproduced and numbered; authors’ responses are printed as intended).

**Q1. When submitting your revision, we need you to address these additional requirements. Please ensure that your manuscript meets PLOS ONE's style requirements, including those for file naming. The PLOS ONE style templates can be found at**

**https://journals.plos.org/plosone/s/file?id=wjVg/PLOSOne_formatting_sample_main_body.pdf and**

[**https://journals.plos.org/plosone/s/file?id=ba62/PLOSOne_formatting_sample_title_authors_affiliations.pdf**](https://journals.plos.org/plosone/s/file?id=ba62/PLOSOne_formatting_sample_title_authors_affiliations.pdf)

**Authors’ response:** We thank the Academic Editor for their comment. We rechecked the PLOS ONE sample templates and made necessary changes to the manuscript to meet the requirements to detail. Affiliations were reformatted to single space, as advised in the PLOS ONE Title, Author, Affiliations Formatting Guidelines (lines 5–11). The Fig 1 caption now appears directly after the paragraph in which it is first cited, according to the PLOS ONE's style requirements as stated in the PLOS ONE style templates (line 197) The location in the previous manuscript version was after Table 10, but since Fig 1 is cited in the paragraph before Table 10, the relocation is justified. The citation style was modified to fit the citation criterion as stated in the PLOS ONE Submission Guidelines. The number of authors listed before “et al” was increased from three to six authors, when applicable. Unwanted information, such as citing date and URLs, were removed. The page number format was changed from minimal to expanded. The reference list was regenerated to apply the changes made to the citation style (line 334).

**Q2. We note that the grant information you provided in the ‘Funding Information’ and ‘Financial Disclosure’ sections do not match. When you resubmit, please ensure that you provide the correct grant numbers for the awards you received for your study in the ‘Funding Information’ section.**

**Authors’ response:** We thank the Academic Editor for their comment. The correct grant numbers were added to the Financial Disclosure Statement and Funding Information sections, where applicable. All funders did not use grant numbers. The information in the Financial Disclosure Statement was checked to match the information in the Funding Information section.

January 22nd, 2024
Manuscript: PONE-D-23-35599
Title: Toll-like receptors 1–9 in small bowel neuroendocrine tumors – Clinical significance and prognosis

**Authors’ responses to Reviewers’ comments** (Reviewers’ comments are reproduced and numbered; authors’ responses are printed as intended).

**Q1. The manuscript (PONE-D-23-35599) entitled Toll-like receptors 1–9 in small bowel neuroendocrine tumors – Clinical significance and prognosis' has been carefully reviewed as per the guidelines of the journal. In my point of view, the manuscript is judged to be of potential interest to the readers and the scientific community as well. While the overall concept of the paper is reasonably satisfactory, it falls within the scope of the journal. However, it has some corrections. The manuscript still has a number of flaws, which need to be addressed before it is formally accepted for publication. For instance, there are some errors in the manuscript's scientific, typographical, and grammatical content. Overall, this manuscript may offer important contributions to the literature, but needs some minor corrections for possible publication in this journal**

**Authors’ response:** We thank the Reviewer for their comment. We identified no specific correction suggestions in this comment but by complying to all the other comments we made sure that the revised version of the manuscript is a better version in terms of scientific, typographical and grammatical content.

**Q2. Title is Ok**

**Authors’ response:** We thank the Reviewer for their comment.

**Q3. Abstract: the abstract is factual concise and explain briefly the purpose of the research. However it would be better to add some key results related insights in the section as well. Also I would recommend suggesting some implications from the finding to represent future perspectives of the study.**

**Authors’ response:** We thank the Reviewer for their comment. To add key results related insight and to represent future perspectives of the study, the section “The current research has future perspective, as it can help create base for clinical drug trials to target specific TLRs with agonists or antagonists to treat neuroendocrine tumors” (lines 30–32) was added to the Abstract section of the manuscript.

**Q4. Introduction: the introduction part is written beautifully, precise and meet the theme of the work.**

**Authors’ response:** We thank the Reviewer for these positive comments.

**Q5. Methods: This section is also written very clear.**

**Authors’ response:** We thank the Reviewer for their comment.

**Q6. Results: All results are ok**

**Authors’ response:** We thank the Reviewer for their comment.

**Q7. Discussion: this part is not very clear, the author should touch every part of the results and compare results with already published work with solid reasons.**

**Authors’ response:** We thank the Reviewer for their comment. The role of TLRs in NETs is largely unknown and research around the topic is scarce. We revisited the literature to bring more comparisons with already published work, as proposed by the Reviewers. The section “Previous NET studies have investigated TLRs mostly in Merkel cell carcinoma (MCC), an aggressive, rare neuroendocrine skin malignancy often caused by Merkel cell polyomavirus (MCV). A study by Jouhi et al characterized TLR 2, 4, 5, 7 and 9 expressions in 128 patients with MCC. [28]. They found preliminary association with TLR expression and clinicopathological variables, such as MCV status (positive/negative), tumor size and patient age. Like their findings, we also found high TLR4 expression to correlate with older age. In 2023, Imon et al used computational formulation to create a multiepitope candidate vaccine against MCV, incorporating TLR4 agonist as an adjuvant for highest immune response activation. [29]. The candidate vaccine was tested in silico, where it seemed to promote immune memory development and natural immune protection against MCV. A pilot study by Bhatia et al investigated intratumoral injections of G100, a TLR4 agonist, in patients with MCC [30]. The patients showed activation of pro-inflammatory changes, which associated with tumor regression. Sample size in the study was small and they found no correlation between clinical responses and baseline TLR expression.” was added to lines 271–282 and the section “A recent 2023 study by Çelik et al on breast cancer found high TLR9 expression to associate with invasive carcinoma and low expression to associate with increased overall survival [32].” was added to lines 289–291 of the Discussion section.

**Q8. Conclusion: In conclusion please summarize the study’s main findings or insights you want to convey as a result of this MS. The limitations and strengths should primarily be highlighted, which could be very helpful in determining the future direction in this field of research direction.**

**Authors’ response:** We thank the Reviewer for their comment. To highlight the strengths and limitations of the study in the Conclusions section, the following section “Confirmatory studies are warranted especially regarding the observed survival association” was replaced with the section “Due to large cohort size, the use of whole section slides and complete registry data, the results are strong, yet preliminary. To determine the future direction in this field of research, confirmatory studies with even larger cohorts and inclusion of G3/NEC disease are warranted – especially regarding the observed survival association.” (lines 324–327).

**Q9. Tables: In the tables what does mean by class M, N and class T? You would better explain it the methods and results.**

**Authors’ response:** We thank the Reviewer for their comment. The explanation of T, N, M (Tumor, Node, Metastasis) was added in Materials and methods subparagraph titled Patients and data collection by replacing the term “TNM” with “Tumor, Node, Metastasis (TNM)” (line 93). The explanation of T-, N- and M-classes were also added in the footnotes of Tables 1–10 (lines 158, 162, 166, 170, 174, .178, 182, 186 and 190).

**Q10. Figures: Figures 1 part B, C, E and F are not clear, please provide high resolution clear images.**

**Authors’ response:** We thank the Reviewer for their comment. The individual images of TLR staining examples were taken in full resolution screen captures with QuPath software, using digitally scanned microscopic whole section images. The screen captures were cropped to include representative tumor areas and combined to a single file to form Fig 1. The file size of the initial Fig 1 was 28.8 MB with excellent image quality. Using the PACE correction, the image size was reduced to one third of the original, 10.2 MB, affecting the image quality as well. We reuploaded Fig 1 as two separate files, labelled Fig1a.tif (9.7 MB) and Fig1b.tif (9.62 MB) resulting in notably better image quality.

**Q11. References: Crosscheck all references and arrange them in accordance with the guidelines of the journal.**

**Authors’ response:** We thank the Reviewer for their comment. All references were crosschecked and edited to meet the formatting criteria provided in the PLOS ONE Submission Guidelines (lines 334–445).
