# Supplementary material for: Toll-like receptors 1–9 in small bowel neuroendocrine tumors–Clinical significance and prognosis
Source: PLoS One. 2024 May 6;19(5):e0302813. doi: 10.1371/journal.pone.0302813 (PMC11073674; doi:10.1371/journal.pone.0302813)
Supplement: Attachment — Submitted filename: Reviewer Comments.docx [file pone.0302813.s004.docx]

**Reviewer :** Revision of the manuscript “Toll-like receptors 1–9 in small bowel neuroendocrine tumors – Clinical significance and prognosis” has been reviewed. There is no further changes to make in the manuscript. Reviewer is pleased to recommend this manuscript for publication.
